# Comparative Analysis of the Chloroplast Genomes of the *Melliodendron* (Styracaceae) Species: Providing Insights into Molecular Evolution and Phylogenetic Relationships

**DOI:** 10.3390/ijms26010177

**Published:** 2024-12-28

**Authors:** Wei Dai, Haozhi Zheng, Menghan Xu, Xingli Zhu, Hui Long, Xiaogang Xu, Yanming Fang

**Affiliations:** 1 Co-Innovation Center for Sustainable Forestry in Southern China, College of Life Science, Nanjing Forestry University, Nanjing 210037, China; weidai@njfu.edu.cn (W.D.); zhenghaozhi@njfu.edu.cn (H.Z.); xumenghan2022@163.com (M.X.); 15287963520@163.com (X.Z.); longhui990313@163.com (H.L.); 2 State Environmental Protection Scientific Observation and Research Station for Ecology and Environment of Wuyi Mountains, Nanjing 210037, China

**Keywords:** *Melliodendron*, chloroplast genome, adaptive evolution, selection pressure, phylogenetic conflict

## Abstract

*Melliodendron xylocarpum* is a member of the Styracaceae family, which is well-known for its remarkable ornamental and medicinal properties. In this research, we conducted comparative analysis of the chloroplast genomes from four samples of *M. xylocarpum,* representing *Melliodendron*. The results demonstrated that the chloroplast genome of four *M. xylocarpum* samples ranging from 157,103 bp to 158,357 bp exhibited a typical quadripartite structure, including one large single-copy (LSC) region (90,131 bp to 90,342 bp), one small single-copy (SSC) region (18,467 bp to 18,785 bp), and two inverted repeat regions (IRs) (24,115 bp to 24,261 bp). Different levels of expansion and contraction were observed in the IR region of four *M. xylocarpum* samples. Besides, *accD* and *ycf1* have been identified under positive selection, potentially linked to the adaptive response of *Melliodendron* to various environmental changes. Conflicting phylogenetic relationships were identified among various genera within the Styracaceae family in the phylogenetic tree constructed using CDS sequences and complete chloroplast genomes. Furthermore, the significance of a large sample size was also highlighted in this study for enhancing the accuracy of findings from phylogenetic analyses. The findings of this research will provide significant insights for future investigations into the evolutionary trends and conservation of the *Melliodendron* species.

## 1. Introduction

It is widely acknowledged that the chloroplast is a crucial participant in the production of energy and biomass in green plants, playing an active role in the process of photosynthesis [1,2]. The chloroplast genome of plants is a typical quadripartite structure, including a large single-copy (LSC) region and two inverted repeat regions (IRs) separated by a small single-copy (SSC) region, all of which exhibit significant levels of conservation [3]. Most chloroplast genomes usually range in size from 120 to 160 kb, encompassing approximately 110–130 uniquely encoded genes [4]. Additionally, almost all chloroplast genomes display a high degree of conservation in both gene content and arrangement, with the majority of genes playing crucial roles in essential functions such as photosynthesis, transcription, and translation [5,6]. Due to the low rate of molecular evolution and single parental inheritance of chloroplast genomes, they have been extensively used in taxonomic identification, phylogeny, genetics, and evolutionary research [7,8]. Moreover, with the rapid advancement of high-throughput sequencing technologies, the application of chloroplast genomes is becoming increasingly prevalent in the field of evolutionary biology [9,10,11].

The diversity of chloroplast genomes provides important resources for revealing phylogenetic relationships across different aspects [12,13,14], and their divergences with nuclear phylogenetic relationships may provide a valuable understanding of the mechanisms behind speciation [15]. Furthermore, environmental changes in the habitat may lead to variations in the genetic composition of chloroplast genomes, which can improve species’ adaptation to specific environments and optimize survival strategies [16,17]. Genes that have experienced positive selection usually have advantages in individual adaptability and reproductive potential [18]. As a result, there has been a notable focus on examining the influence of selective pressure and adaptive evolution in molecular research, establishing a solid foundation for the exploration of germplasm resources.

The Styracaceae family consists of 12 genera and over 160 species, primarily distributed in Asian regions, the Americas, and the Mediterranean [19]. *Melliodendron xylocarpum* Hand.-Mazz. is a member of the Styracaceae family, which is well-known for its remarkable ornamental and medicinal properties. It is a rare species endemic to China and primarily distributed in the southern regions of the country, such as Guang Dong, Guang Xi, and Si Chuan province [20]. Researchers have identified *Melliodendron jifungense* Hu and *Melliodendron wangianum* Hu as a synonym of *M. xylocarpum* [19,20]. Hence, *M. xylocarpum* represents the sole species within the genus *Melliodendron*. At present, the study of *M. xylocarpum* is relatively scarce, with only two samples of chloroplasts and their sequencing available [21,22]. However, this is insufficient for a rare species like *M. xylocarpum*. During the field investigation, it was discovered that the morphology of *M. xylocarpum* varied significantly, such as in leaf size and color, etc. This situation is similar to that of *S. japonicus* in Styracaceae [15]. In other words, there is considerable genetic diversity within the same species. Therefore, genome analysis has emerged as an important approach to better explore the evolutionary history of *M. xylocarpum* and to formulate biodiversity conservation strategies.

In this study, we collected and sequenced four typical *M. xylocarpum* with distinct distributions in China and carried out an integrated analysis with *M. xylocarpum* that had been published in NCBI. The ultimate objectives of this study are to (1) characterize the structural features of the chloroplast genome in *M. xylocarpum* and analyze the genes linked to adaptive evolution, (2) investigate potentially adaptive evolutionary patterns in *Melliodendron* through the analysis of its associated chloroplast genes, (3) and reconstruct and compare phylogenetic relationships of *Melliodendron* within the Styracaceae family. Through these explorations, a more reliable phylogenetic map of *M. xylocarpum* and its evolutionary path can be acquired. Additionally, adaptive evolutionary analysis allows us to offer potential target genes for conservation and breeding, formulate effective conservation strategies, provide a scientific basis for species identification and germplasm conservation, and enhance species’ adaptive improvement.

## 2. Results

### 2.1. Characteristics of the Chloroplast Genome

**Figure 1:**
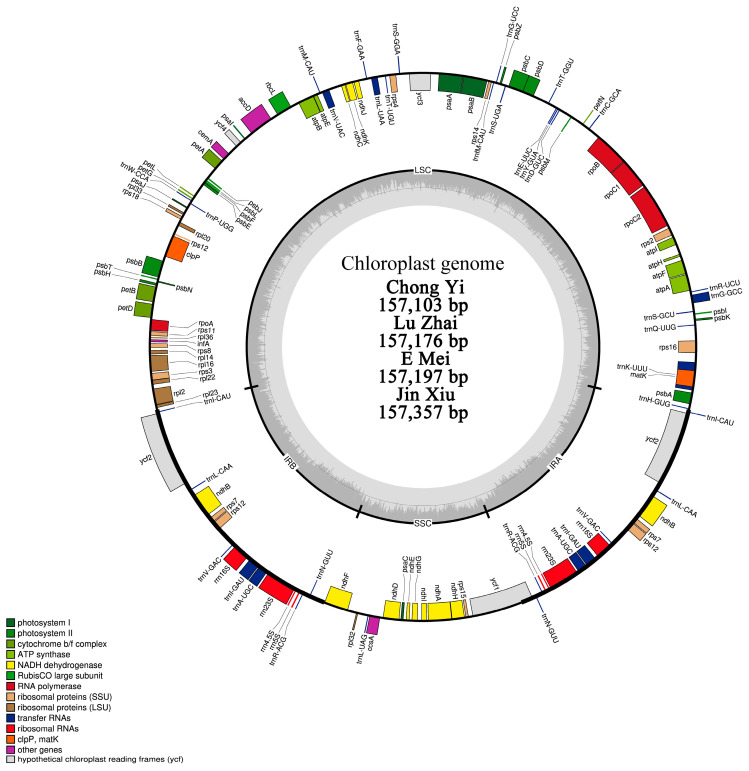
Gene map of four *M. xylocarpum* chloroplast genomes.

After sequencing, annotating, and visualizing by OGDRAW, the chloroplast genome of four *M. xylocarpum* samples exhibited a typical quadripartite structure, including one large single-copy (LSC) region (90,131 bp to 90,342 bp), one small single-copy (SSC) region (18,467 bp to 18,785 bp), and two inverted repeat regions (IRs) (24,115 bp to 24,261 bp) (Figure 1 and Table 1). The full size of the complete genomes varied between 157,103 bp and 158,357 bp, and the GC content ranged from 36.9% to 37% (Table 1). Each chloroplast genome consisted of a total of 130 genes, which comprised 8 rRNA genes, 37 tRNA genes, and 85 protein-coding genes (Table 1). All genes can be divided by functions into three categories: (1) photosynthesis genes; (2) self-replication genes; (3) and other genes (Table 2).

### 2.2. Analysis of the Chloroplast Genome of Melliodendron

**Figure 2:**
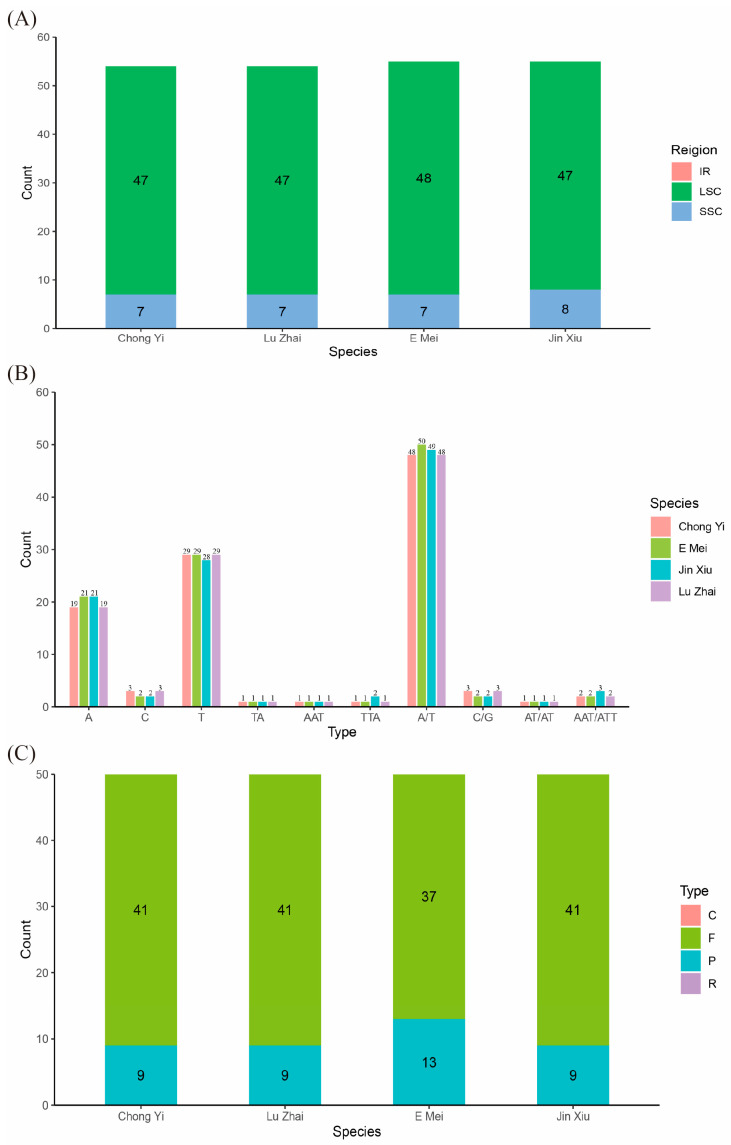
Analysis of SSR sites and repetitive sequences in four chloroplast genomes. (**A**): Distribution of SSRs in the four samples within three regions of the complete chloroplast genome; (**B**): Number of different SSRs loci types; (**C**): The number of four distinct repeat types. Note: In (**C**), C: complementary repeats, F: forward repeats, P: palindromic repeats, R: reverse repeats.

According to the analysis of SSR, the chloroplast genome of *Melliodendron* possesses 50–54 SSRs, and most of them were found in the LSC region. A few SSRs were located in the SSC region, and no SSR was found in the IR region (Figure 2A). Three different types of SSRs were identified in the four species, including mononucleotide (A/T, C/G), dinucleotide (AT/AT), and trinucleotide (AAT/ATT), and most of them are mononucleotide (Figure 2B). There were a total of 50 repeats in these four species, encompassing both forward and palindromic repeats (Figure 2C). However, no complementary or reverse repeats were detected in this analysis. Additionally, the forward repeats are the predominant type of repeat found in the four species (Figure 2C).

**Figure 3:**
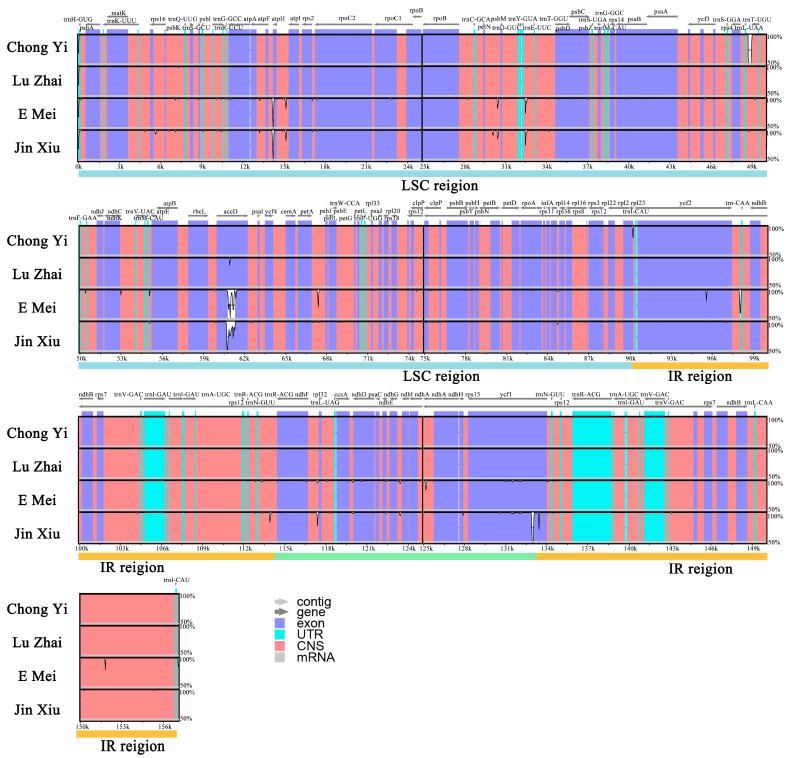
Variation level of chloroplast genome sequences of four *Melliodendron* species. Blue represents coding regions, green represents RNA regions, and red represents non-coding regions. The y-axis indicates the level of variation (between 50 and 100%), and the x-axis represents the coordinate in the chloroplast genome.

The analysis of chloroplast genomes of four *Melliodendron* species were conducted by mVISTA with *M. xylocarpum* (GenBank accession: MN175625) as the reference sequence. The results revealed that the chloroplast genomes of four *Melliodendron* species were highly similar and conserved (Figure 3). However, the most significant variation is observed in the *accD* genes of E Mei and Jin Xiu. Additionally, related mutations were found in *atph*, *ycf1*, and *ycf2*. Moreover, there were also varying degrees of variations in the non-coding region, and it was observed that IR regions exhibited higher levels of conservation than the LSC and SSC regions (Figure 3).

**Figure 4:**
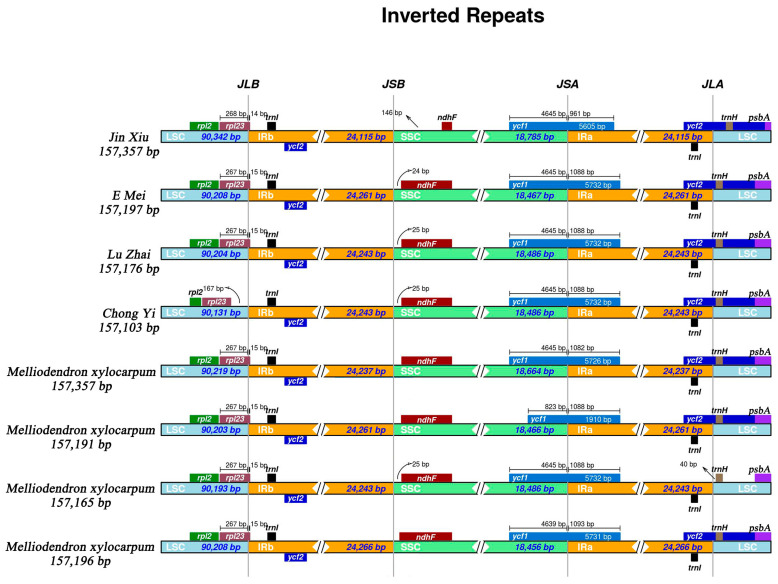
Comparison of the chloroplast genome structure in four *Melliodendron* samples and the other four *Melliodendron* species in IR (inverted repeat), LSC (large single-copy) and SSC (small single-copy) regions.

The comparative analysis of chloroplast genome sequences from four samples of *Melliodendron* revealed different levels of expansion and contraction of the IR region in comparison to the genomes of other *Melliodendron* samples (Figure 4). The gene *rpl23* was located at the junction of LSC-IRB among Jin Xiu, E Mei, and Lu Zhai, while the *rpl23* of Chong Yi has completely crossed into the LSC region. In addition, it was observed that *ndhF* was 24–146 bp away from the junction of IRB-SSC, and *trnH* was completely located at the LSC region in Jin Xiu, E Mei, Lu Zhai and Chong Yi. The junction of SSC-IRA was identified within the *ycf1* gene, and *ycf2* was located at the junction of IRA-SSC (Figure 4).

### 2.3. Codon Bias Analysis and Selective Pressures in the Evolution

**Figure 5:**
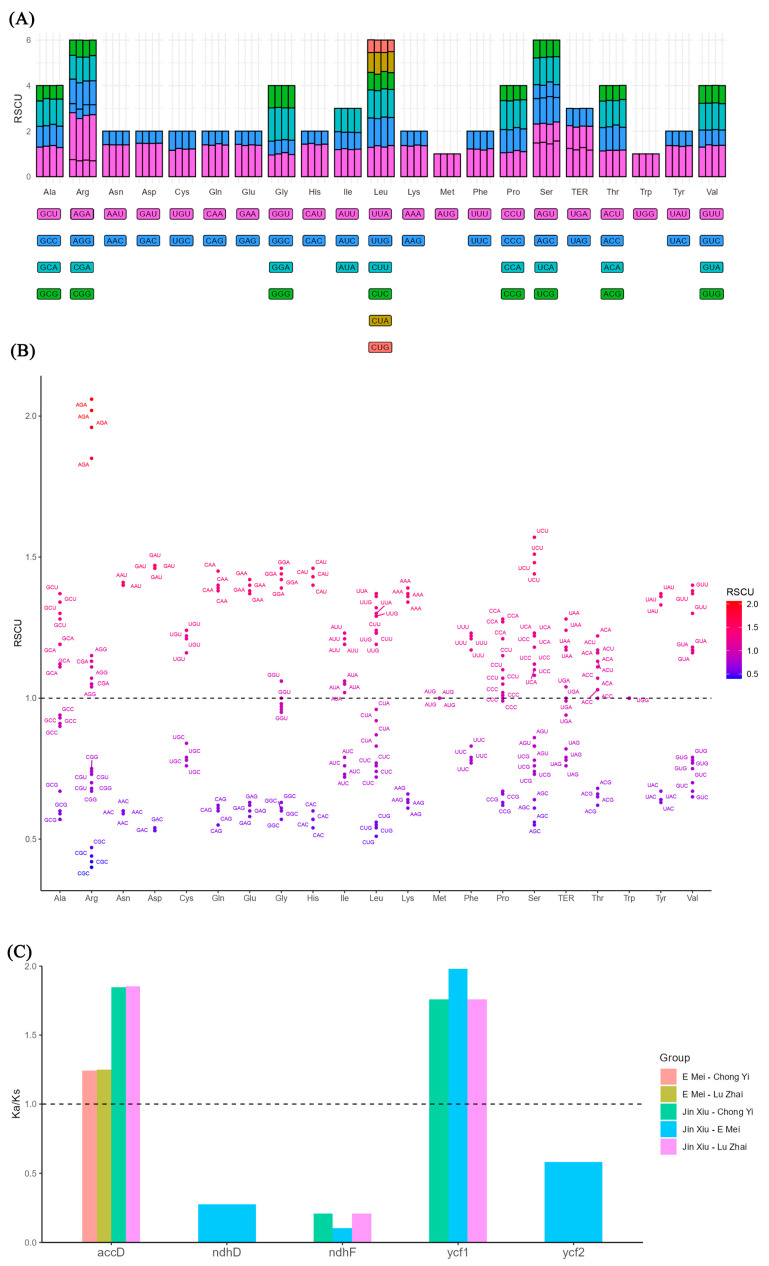
Relative synonymous codon usage and selective pressures in the evolution. (**A**): Codon content of 20 amino acids and stop codons in all protein-coding genes of four *Melliodendron* chloroplast genomes; (**B**): Distribution of codon preference in four *Melliodendron* species; (**C**): Ka/Ks values of protein-coding genes of the four comparative combinations. Note: in (**A**), the top panel shows the RSCU for the corresponding amino acids, the colored blocks which are shown below represent different codons; In (**C**), Ka: nonsynonymous; Ks: synonymous.

The relative synonymous codon usage (RSCU) values showed a high degree of similarity among the chloroplast genomes of the four *Melliodendron* species (Figure 5A). A total of 22 codons showed an RSCU value exceeding 1 (Figure 5B), and just two of them ended in G (AGG and UUG). Additionally, it was observed that the RSCU value of 19 codons was lower than 1, with 10 terminating in G and 9 terminating in C.

Since certain genes exhibited Ks or Ka values of 0, the majority of Ka/Ks ratios were considered to be invalid. Therefore, the evaluation of Ka/Ks was limited to just four genes with valid values out of the 78 common genes (Figure 5C). The results revealed that the Ka/Ks ratios of two genes (*accD* and *ycf1*) in the four *Melliodendron* species were found to be greater than 1.

### 2.4. Nucleic Acid Polymorphism Analysis

**Figure 6:**
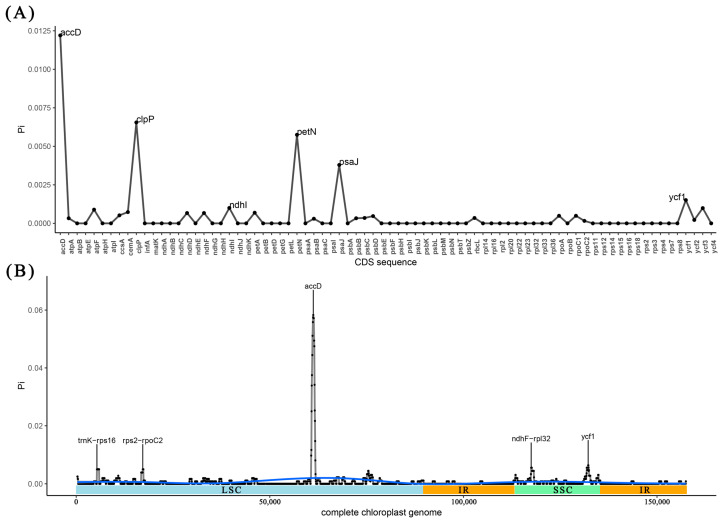
Nucleotide diversity of chloroplast genomes in four *Melliodendron* species. (**A**): Pi in CDS; (**B**): chloroplast genome Pi value. Note: window length: 600 bp, step length: 50 bp; X axis: position of the midpoint of each window; Y axis: Pi of each window. The blue line represents the trajectory of the value of Pi.

The analysis of Pi value in CDS sequences revealed that *accD*, *clpP*, *petN*, *psaJ*, *ycf1*, and *ndhI* showed elevated Pi values among the 80 CDS sequence genes (Figure 6A). Additionally, upon analyzing the Pi value in complete chloroplast genome sequences, it was observed that two gene regions (*accD* and *ycf1*) as well as three intergenic regions (*ndhF-rpl32*, *trnK-rps16*, *rps2-rpoC2*) exhibited high Pi values (Figure 6B). Furthermore, the analysis of the complete chloroplast genome sequences also demonstrated that there was greater diversity in the SSC and LSC regions when compared to the IR regions (Figure 6B).

### 2.5. Phylogenetic Analysis

**Figure 7:**
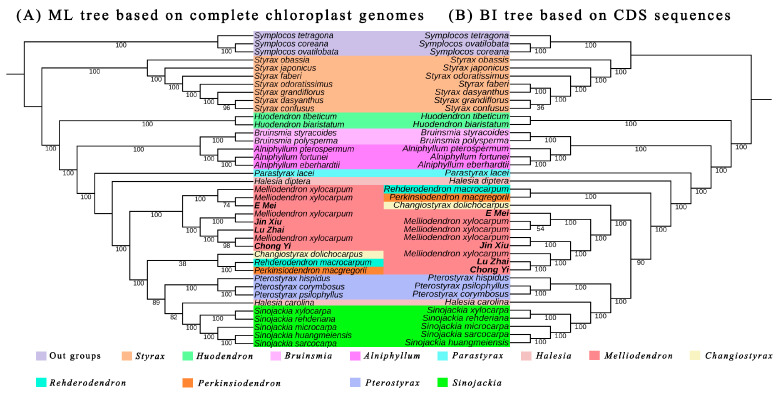
Phylogenetic tree analysis using maximum likelihood (ML) and Bayesian inference (BI) based on complete chloroplast genomes and CDS sequences. (**A**) ML tree based on complete chloroplast genomes. (**B**) BI tree based on CDS sequences. The numbers at nodes represent bootstrap values per 1000 replicates.

The complete chloroplast genome and CDS sequence were utilized to reconstruct the phylogenetic relationships among *Melliodendron* and 11 other genera within the Styracaceae, employing maximum likelihood (ML) and Bayesian inference (BI) methods. Both the ML and BI trees demonstrated a substantial level of similarity, despite the presence of differing degrees of phylogenetic conflict (Figure 7).

In the ML tree, all *Melliodendron* species collectively formed a monophyletic clade that was sister to *Changiostyrax*, *Rehderodendron*, *Perkinsiodendron*, *Pterostyrax*, *Halesia*, and *Sinojackia*. However, *Melliodendron* formed a monophyletic group with *Changiostyrax,* and it was sister to *Pterostyrax*, *Halesia*, and *Sinojackia* in the BI tree. Additionally, the position of *Styrax*, *Huodendron*, *Bruinsmia*, *Alniphyllum*, *Parastyrax*, *Halesia*, *Pterostyrax*, and *Sinojackia* remained consistent in both the ML tree and BI tree. There were evident phylogenetic conflicts between *Halesia diptera* and *Halesia carolina*. *Halesia diptera* formed a single clade in both the ML tree and BI tree, while *Halesia carolina* composed into a monophyletic group with *Sinojackia*. Moreover, the support rates for certain genera within *Styrax*, *Melliodendron*, and *Changiostyrax* are lower in both the ML tree and BI tree.

## 3. Discussion

In this research, we performed sequencing of four samples of *M. xylocarpum* and carried out a comparative analysis of their chloroplast genomes. The results indicate that there is a significant resemblance in the structure of their chloroplast genomes, but identifiable mutation hotspots still exist. Particularly, (1) different levels of expansion or contraction are present in the IR region of four chloroplast genomes of *M. xylocarpum*; (2) positive selection was identified in two genes, including *ycf1* and *accD*; (3) phylogenetic trees derived from both coding sequences (CDS), and complete chloroplast genomes revealed multiple conflicts in the inference of relationships. Hence, we will discuss these three points in the subsequent sections.

### 3.1. Expansion and Contraction of IR Region

The results of this research indicate that the boundary genes in all *Melliodendron* samples are constantly conserved without notable changes, whereas, the IR regions of Chong Yi, Lu Zhai, E Mei, and Jin Xiu exhibit different levels of expansion and contraction when compared to other samples of *Melliodendron*. Although these genetic alterations involve only a few bp, it is worthy of noting that such changes can arise in the same species.

A variety of factors may influence the expansion or contraction of IR regions, such as genome rearrangement, evolutionary pressures, random mutations, and gene transfer [23]. Although the IR region is often regarded as highly conserved [24], variation in boundary genes is a common phenomenon in the process of chloroplast genome evolution. In terms of function, the expansion and contraction of the IR region directly result in changes in the copy number of related genes and influence the expression level of genes. Meanwhile, boundary alterations can give rise to the production of pseudogenes, affecting the function and stability of the corresponding genes [25,26].

The expansion of the IR region can contribute to improving the genetic stability of the whole genome [27,28]. On the contrary, the contraction of the IR region could result in a higher level of genetic variation across the entire genome. In this research, we observed that both contraction and expansion of the IR region occurred within the chloroplast genomes of *Melliodendron*, suggesting that their genomes are undergoing dynamic changes [25]. Regarding evolution, the expansion and contraction of the IR region are common occurrences during the evolution of chloroplast genomes in plants, and they might be related to the adaptation and differentiation of species. Generally, changes in the IR region can affect the results of phylogenetic analysis, and the differences in the IR region between different species can reflect evolutionary relationships and the differentiation history. At present, the chloroplast genomes of several species of *Styrax* in Styracaceae show varying degrees of expansion and contraction [29]. Among them, the representative species of the genus, *S. japonicus*, presented heterogeneity in the IR region among species [15]. In contrast, *S. oblongicarpa* from *Sinojackia* indicates that the chloroplast genome is highly conserved, and the length of the IR region is completely consistent [30]. However, in this research, *M. xylocarpum* is the sole species of *Melliodendron*, which indicates that different populations within this genus are undergoing differentiation. This will directly result in the formation of new species and genetic diversity in *Melliodendron*. Thus, different populations are following individual evolutionary paths.

### 3.2. Positive Selection of accD and ycf1

In our research, we identified that *accD* and *ycf1* of four *Melliodendron* samples exhibited positive selection. The positive selection of *accD* and *ycf1* may indicate an improvement of gene function in response to environmental pressures [31,32].

The *accD* gene is responsible for encoding a crucial component of the acetyl-CoA carboxylase (ACCase) complex, which plays a pivotal role in the initiation of fatty acid synthesis in plants [33,34]. For instance, the improvement of *accD* expression resulted in a substantial augmentation of ACCase abundance within plastids, leading to a notable increase in fatty acid content in tobacco leaves [35]. Kode et al. found that the *accD* gene is crucial in promoting leaf growth and preserving plastid compartment in tobacco [36]. Bryant et al. also demonstrated that the presence of *accD* is indispensable during the embryonic developmental stage in Arabidopsis [37]. Furthermore, previous research has indicated that the positive selection of the *accD* gene suggests it may be undergoing adaptive evolution to better align with the plant’s requirements, potentially in response to environmental stresses or for improving its photosynthetic efficiency [33]. Hence, the positive selection of *accD* genes in four *Melliodendron* samples may have significant implications for the adaptive evolution of *Melliodendron*, particularly in its response to environmental pressures.

The *ycf1* gene is the second largest gene in chloroplasts, responsible for encoding a protein consisting of approximately 1800 amino acids [38]. Prior studies have indicated that *ycf1* is crucial for plant survival, maintaining the stability of photosynthesis complexes, and functioning as a component of the Tic complex in Arabidopsis [39,40]. The positive selection of the *ycf1* gene has also been observed in other species, including *Citrus*, *Oenothera*, and *Cardamine* [41,42,43]. Jiang et al. and Choi et al. suggested that the positive selection of the *ycf1* gene in certain groups indicates its potential adaptive evolution to better accommodate the plant’s needs [44,45]. Consequently, the positive selection of the *ycf1* gene in *Melliodendron* indicates its important role in controlling chloroplast gene expression or adapting to environmental changes.

Additionally, the positive selection of *accD* and *ycf1* may be attributed to the plants’ adaptation to abiotic stress factors, including fluctuating temperatures, limited water availability, and high salinity levels [46,47,48]. From the perspective of evolution, *accD* and *ycf1* subjected to positive selection indicates the plant’s enduring adaptation to the environment. This long-term process may encompass speciation, adjustments within ecological niches, and the formation of biodiversity [49,50].

To sum up, the positive selection *accD* and *ycf1* genes might have been formed as a result of the influence of diverse climatic environments and geographical patterns, offering a novel strategy for genetic enhancement and environmental adaptation. For instance, by enhancing the expression of the *accD* gene, the plant’s fatty acid synthesis capacity is improved, thereby increasing its nutritional and energy value. The study of the *ycf1* may assist in protecting and optimizing photosynthesis efficiency, which holds significant implications for enhancing crop yields and adapting to climate change. Additionally, positive selection of these genes might also provide clues for understanding how plants adapt to environmental changes during long-term evolution, which has profound implications for the conservation of biodiversity and ecosystem stability.

### 3.3. Phylogenetic Analysis

Through phylogenetic analysis, it is evident that the positions of *Melliodendron* and 11 other genera within the Styracaceae demonstrate a relative stability, despite some conflicts observed in both the ML tree and BI tree. The conflicts of phylogenetic relationships between the ML tree and BI tree can be attributed to various factors.

Generally, phylogenetic conflicts arise from six main reasons. (1) Gene duplication and loss. Gene duplication events can generate multiple copies, which can be lost in different manners in different species, causing the gene tree to be inconsistent with the species tree [51]; (2) Horizontal gene transfer (HGT). HGT between chloroplast genomes can influence the evolutionary process of plant cytoplasmic genetic material, enhance the interconnection among genomes, and promote the evolution of plant populations, which may also result in phylogenetic conflicts [52]; (3) Hybridization. Chloroplast genomes may exhibit maternal or paternal inheritance patterns during hybridization, which can lead to conflicts in the construction of phylogenetic trees [53]; (4) Incomplete Lineage Sorting (ILS). ILS is an important factor in plant chloroplast genomes, as it can cause inconsistent phylogenetic signals across different gene loci, leading to conflicts [54]; (5) Long-branch attraction. Due to potential differences in evolutionary rates within chloroplast genomes, rapidly evolving lineages might be incorrectly grouped together because of inappropriate model assumptions, leading to misjudgments in phylogenetic relationships [55]; (6) Sampling bias and insufficient data. In studies of chloroplast genomes, insufficient sample numbers or incomplete data can increase the uncertainty of phylogenetic analysis, resulting in conflicts [56].

In summary, phylogenetic conflicts consist of soft conflicts and hard conflicts. Soft conflicts arise from the inappropriate selection of analytical models, sampling bias, and insufficient data. In contrast, hard conflicts result from biological processes, namely, the inherent variations in genomes and coding sequence (CDS) regions.

In this study, both hard and soft conflicts were identified in the phylogenetic analysis. In *Melliodendron*, the support rate for one or two branches was relatively low, and the relationships within this genus were inconsistent. These two points indicate that the current phylogenetic map is incomplete. Therefore, more samples and populations are needed to complete the evolutionary map. Once these data are obtained, DNA barcodes, including specific ones, could be identified for use in screening germplasm resources and breeding varieties. Furthermore, in future research, the sequencing of nuclear genomes, the application of advanced Bayesian methods, and the implementation of customized multi-dimensional layouts could enhance the accuracy and resolution of the phylogenetic analysis.

## 4. Materials and Methods

### 4.1. Plant Samples, Genomic DNA Extraction and Genome Sequencing

**Figure 8:**
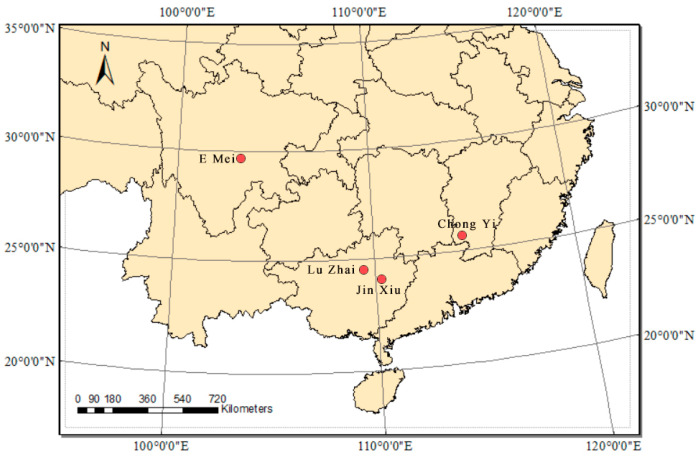
The red circles represent the location of four leaf-sampling sites (E Mei, Lu Zhai, Jin Xiu, Chong Yi).

In this research, four fresh-leaf samples originate from four typically natural populations with diverse distributions and have a certain range in terms of geography and distance in China (Figure 8), including (Chongyi city, Jiangxi province) (25.69° N, 114.31° E), (Luzhai city, Guangxi province) (24.49° N, 109.74° E), (Emeishan city, Sichuan province) (29.52° N, 103.33° E), and (Jinxiu city, Guangxi province) (24.14° N, 110.18° E). We collected them from April 2022 to June 2022, and these samples were named as Chong Yi (CY), Lu Zhai (LZ), E Mei (EM), and Jin Xiu (JX), for short, in order to distinguish them clearly. Then, we utilized the Plant Genomic DNA Kit from Nanjing Genepioneer Biotechnologies Inc. (Nanjing, China) to perform genomic DNA extraction in each leaf sample following the instructions of the manufacturer. A Nandrop 2000 instrument (Thermo Fisher Scientific, Waltham, MA, USA) was used for the assessment of DNA concentration and purity. Agarose gel electrophoresis was employed to assess the integrity of the DNA. In accordance with Illumina’s standard procedure, libraries were prepared using the total DNA extracted and subsequently sequenced on the Illumina NovaSeq 6000 platform (Illumina, San Diego, CA, USA), utilizing a sequencing read length of 150 bp. The complete extraction and sequencing of DNA were carried out by Nanjing Genepioneer Biotechnologies Inc. (Nanjing, China).

### 4.2. Chloroplast Genome Assembly, Annotation and Sequence Analysis

CLC Genomics Workbench v9 was used to trim the raw reads in order to obtain clean data. Then, the resultant clean data were assembled by SPAdes v4.0.0 (https://microbialgenomicslab-spring2022.readthedocs.io/en/latest/GenomeAssemblies.html, accessed on 1 July 2024) [57] to acquire seed sequences of chloroplast genomes, and the iterative k-mer extend seeds were used to obtain the contig sequences. SSPACE v2.0 [58] was used to link the contig sequences into scaffolds, and then the gaps were filled by Gapfiller v2.1.1 [59].

We conducted a comparison of the assembled sequences using Blast (https://blast.ncbi.nlm.nih.gov/Blast.cgi, accessed on 1 July 2024) against the NCBI database in order to determine the most appropriate reference genome to guarantee the precision of annotation [60]. Then, CPGAVAS2 (http://47.96.249.172:16019/analyzer/home, accessed on 1 July 2024) [61] and GeSeq (https://chlorobox.mpimp-golm.mpg.de/geseq.html, accessed on 1 July 2024 ) [62] were employed to annotate chloroplast genomes. Geneious was used to refine and manually correct the annotated chloroplast genome sequences to improve the accuracy of annotation results. The circular chloroplast genome maps were visualized by the online tool OGDRAW (https://chlorobox.mpimp-golm.mpg.de/OGDraw.html, accessed on 1 July 2024) [63]. The assembled chloroplast genome sequences have been deposited in the NCBI database (accession number: SAMN43636518, SAMN43645341, SAMN43648579, SAMN43651081).

The identification of forward, reverse, palindromic, and complementary repeats within the chloroplast genome were conducted by the online tool REPuter (https://bibiserv.cebitec.uni-bielefeld.de/reputer, accessed on 1 July 2024) [64]. The Perl script called MISA v2.1 [65] was employed to identify Simple sequence Repeats (SSRs), such as mono-, di-, tri-, tetra-, penta-, and hexa-nucleotides with minimum thresholds established at 10, 6, 5, 5, 5, and 5, correspondingly.

### 4.3. Analysis of Codon Usage Bias and Selective Pressures in the Evolution

The program PhyloSuite [66] was utilized for the extraction of full-length CDS sequences and their concentration. Then, we used CodonW v1.4.2 [67] for the calculation of parameters that indicate bias in codon usage, such as nucleotide compositions at the third position (A3s, U3s, and G3s), GC content at third codon positions (GC3s), codon adaptation index (CAI), codon bias index (CBI), effective number of codons (ENC), and relative synonymous codon usage (RSCU).

Mafft v7.520 [68] was employed for the extraction and alignment of 78 common protein coding sequences in order to calculate the substitution rates of synonymous (Ks) and non-synonymous (Ka), as well as their respective ratios (Ka/Ks values). The computation of Ka/Ks values and selection pressure were conducted by KaKs calculator 3.0 [69], and we selected the YN algorithm in KaKs calculator, which is commonly employed in research on evolution.

### 4.4. Comparative Analysis

We utilized the online tool IRscope (http://genocat.tools/tools/irscope.html, accessed on 1 July 2024) [70] to conduct an analysis and comparison of the boundaries within the LSC, SSC, and IR regions among the chloroplast genome sequences of four *M. xylocarpum* and their sibling species previously published in NCBI (Accession number: MG719837; MN175625; MN378563; MZ153072). Furthermore, mVISTA (https://genome.lbl.gov/vista/mvista/submit.shtml, accessed on 1 July 2024) was used to conduct a comparative analysis of the chloroplast genome structure of four *M. xylocarpum* using the Shuffle-LAGAN model (Lawrence Berkeley National Laboratory, Stanford, CA, USA), with *M. xylocarpum* (GenBank accession: MN175625) as the reference sequence [71]. The nucleotide diversity (Pi) of CDS sequences and complete chloroplast genomes was calculated by DNAsp v6.12.03 [72]. The step size was designated as 50 bp and a window length of 600 bp was employed.

### 4.5. Phylogenetic Analysis

In order to investigate the phylogenetic positions of *Melliodendron* in Styracaceae, we utilized the chloroplast genome of 28 Styracaceae samples from 12 different genera, and 2 species belonging to *Symplocos* were designated as outgroup to reconstruct the phylogenetic tree. We employed the MAFFT v.7.520 [68] to align 30 chloroplast sequences, and ModelFinder V2.3.6 [73] was used to determine the best-fit model. The maximum likelihood (ML) analysis was conducted in IQ-tree V1.6.0 [74] using the complete chloroplast genome, with 1000 bootstrap replicates. In addition, the CDS sequence was utilized for the construction of a Bayesian inference (BI) tree through Mrbayes v3.2 [75]. The BI analysis was conducted for a total of 10 million generations, with the sampling of a tree occurring every 1000 generations. The initial 25% of the sampled trees were discarded as burn-in.

## 5. Conclusions

In this research, we sequenced and analyzed the chloroplast genomes from four *M. xylocarpum* samples, representing *Melliodendron*. The primary findings of this study encompass the following four aspects: (1) Different levels of expansion and contraction were observed in the IR region of four *Melliodendron* samples, indicating an evolutionary response to environmental pressures and adaptive evolution to enhance the adaptability of *Melliodendron*. (2) Both *accD* and *ycf1* have been found to be under positive selection, reflecting the adaptive response of *Melliodendron* to diverse environmental changes. (3) Conflicting phylogenetic relationships were observed among different genera within the Styracaceae family, which may be attributed to gene flow, genetic hybridization, gene loss or recombination. (4) This research also emphasized the significance of a substantial sample size in improving the precision of results from phylogenetic analyses.

In conclusion, this research reveals the characteristic complete chloroplast genomes of *Melliodendron* and clarifies its natural distribution patterns. The comparison results not only deepen our understanding of the evolutionary history of this genus but also offer valuable perspectives for conservation efforts. Our findings imply that individuals from populations showing certain adaptive genetic variations could be strategically relocated to areas encountering similar environmental stressors. This approach, known as assisted migration or managed relocation, has the potential to enhance the overall resilience of the species by increasing the genetic diversity of recipient populations and their adaptive capacity to environmental changes. Moreover, the identified genes can have multiple uses, including the development of DNA barcodes for species identification, comprehension of plant tolerance to stress, guidance for variety breeding programs, assistance in the conservation of endangered species, and exploration of the genetic biodiversity within and among species. These applications highlight the practical implications of our research for both basic science and conservation strategies, emphasizing the significance of genomic data in informing effective management actions for rare species like *Melliodendron*.

## Figures and Tables

**Table 1:** Characteristics of assembled chloroplast genome basic structure.

Species	Total Size (bp)	GC Content (%)	LSC Size (bp)	IR Size (bp)	SSC Size (bp)	Protein Coding Genes	rRNA	tRNA
Chong Yi	157,103	38.3	90,131	24,243	18,486	85	8	37
Lu Zhai	157,176	38.2	90,204	24,243	18,486	85	8	37
E Mei	157,197	38.2	90,208	24,261	18,467	85	8	37
Jin Xiu	157,357	38.3	90,342	24,115	18,785	85	8	37

**Table 2:** Genes in different groups and functions of Chong Yi, Lu Zhai, E Mei, and Jin Xiu.

Category of Genes	Function of Genes	Name of Genes	No.
Photosynthesis	Subunits of photosystem I	*psaA*, *psaB*, *psaC*, *psaI*, *psaJ*	5
	Subunits of photosystem II	*psbA*, *psbB*, *psbC*, *psbD*, *psbE*, *psbF*, *psbH*, *psbI, psbJ*, *psbK*, *psbL*, *psbM*, *psbN*, *psbT*	14
	Subunits of NADH dehydrogenase	ndhA *, ndhB * (2), *ndhC*, *ndhD*, *ndhE*, *ndhF*, *ndhG, ndhH*, *ndhI*, *ndhJ*, *ndhK*	12
	Subunits of cytochrome b/f complex	*petA*, petB *, petD *, *petG*, *petL*, *petN*	6
	Subunits of ATP synthase	*atpA*, *atpB*, *atpE*, atpF *, *atpH*, *atpI*	6
Self-replication	Large subunit of rubisco	*rbcL*	1
	Proteins of large ribosomal subunit	*rpl14*, rpl16 *, rpl2 *, *rpl20*, *rpl22*, *rpl23*, *rpl32, rpl33*, *rpl36*	9
	Proteins of small ribosomal subunit	*rps11*, rps12 ** (2)*, rps14*, *rps15*, rps16 *, *rps18, rps19*, *rps2*, *rps3*, *rps4*, *rps7* (2), *rps8*	14
	Subunits of RNA polymerase	*rpoA*, *rpoB*, rpoC1 *, *rpoC2*	4
	Ribosomal RNAs	*rrn16* (2), *rrn23* (2), *rrn4.5* (2), *rrn5* (2)	8
	Transfer RNAs	trnA-UGC * (2), *trnC-GCA*, *trnD-GUC*, *trnE-UUC*, *trnF-GAA*, *trnfM-CAU*, trnG-GCC *, *trnG-UCC*, *trnH-GUG*, *trnI-CAU* (2), trnI-GAU * (2), trnK-UUU *, *trnL-CAA* (2), trnL-UAA *, *trnL-UAG*, *trnM-CAU*, *trnN-GUU* (2), *trnP-UGG*, *trnQ-UUG*, *trnR-ACG* (2), *trnR-UCU*, *trnS-GCU*, *trnS-GGA*, *trnS-UGA*, *trnT-GGU*, *trnT-UGU*, *trnV-GAC* (2), trnV-UAC *, *trnW-CCA*, *trnY-GUA*	37
Other genes	Maturase	*matK*	1
	Protease	clpP **	1
	Envelope membrane protein	*cemA*	1
	Acetyl–CoA carboxylase	*accD*	1
	c-type cytochrome synthesis gene	*ccsA*	1
	Translation initiation factor	*infA*	1
Genes of unknown function	Conserved hypothetical chloroplast ORF	*lhbA*, *ycf1*, *ycf15* (2), *ycf2* (2), ycf3 **, *ycf4*	8
Total number of genes		130	

Note: Gene * means gene with one intron; Gene ** means gene with two introns; Gene (2) means multi-copy genes.

## Data Availability

The original contributions presented in this study are publicly available. These data can be found in the NCBI database (accession number: SAMN43636518, SAMN43645341, SAMN43648579, SAMN43651081).
